# Supplementary figures and images for: RB depletion is required for the continuous growth of tumors initiated by loss of RB
Source: PLoS Genet. 2021 Dec 8;17(12):e1009941. doi: 10.1371/journal.pgen.1009941 (PMC8654178; doi:10.1371/journal.pgen.1009941)

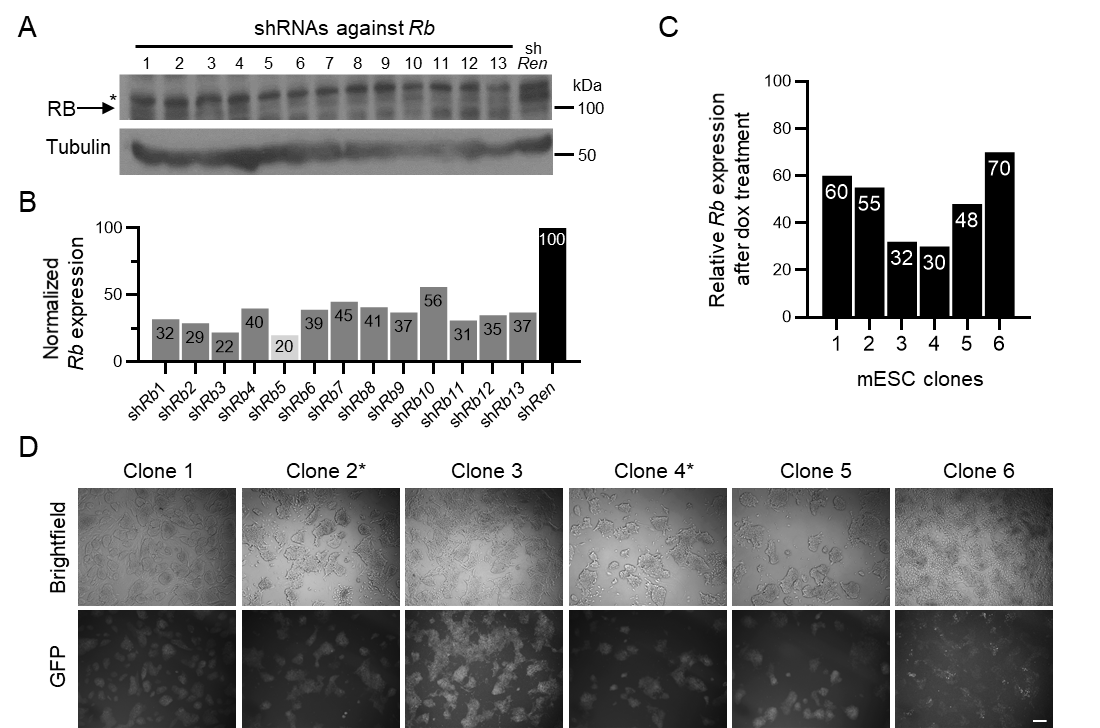

Supplement: S1 Fig — A. Immunoblot analysis on protein extracts from mouse C2C12 cells stably expressing different shRNA against Rb. Tubulin serves as a loading control. *, non-specific band. B. RT-qPCR on RNA isolated from mouse NIH3T3 cells stably expressing different shRNAs against Rb. Expression is normalized to a control shRNA against Renilla luciferase (Ren). C. RT-qPCR analysis of Rb levels in different mouse embryonic stem cells (mESC) clones with inducible shRb5 treated with doxycycline (dox) normalized against untreated controls. D. Representative images of the different mESC clones with inducible shRb5 under dox treatment. * marks clones that were euploid by karyotyping. Scale bar, 100μm. Note the induction of GFP as visualized by fluorescence. Clone shRb5-4 was selected for the generation of mice. (TIF) [file pgen.1009941.s001.tif]

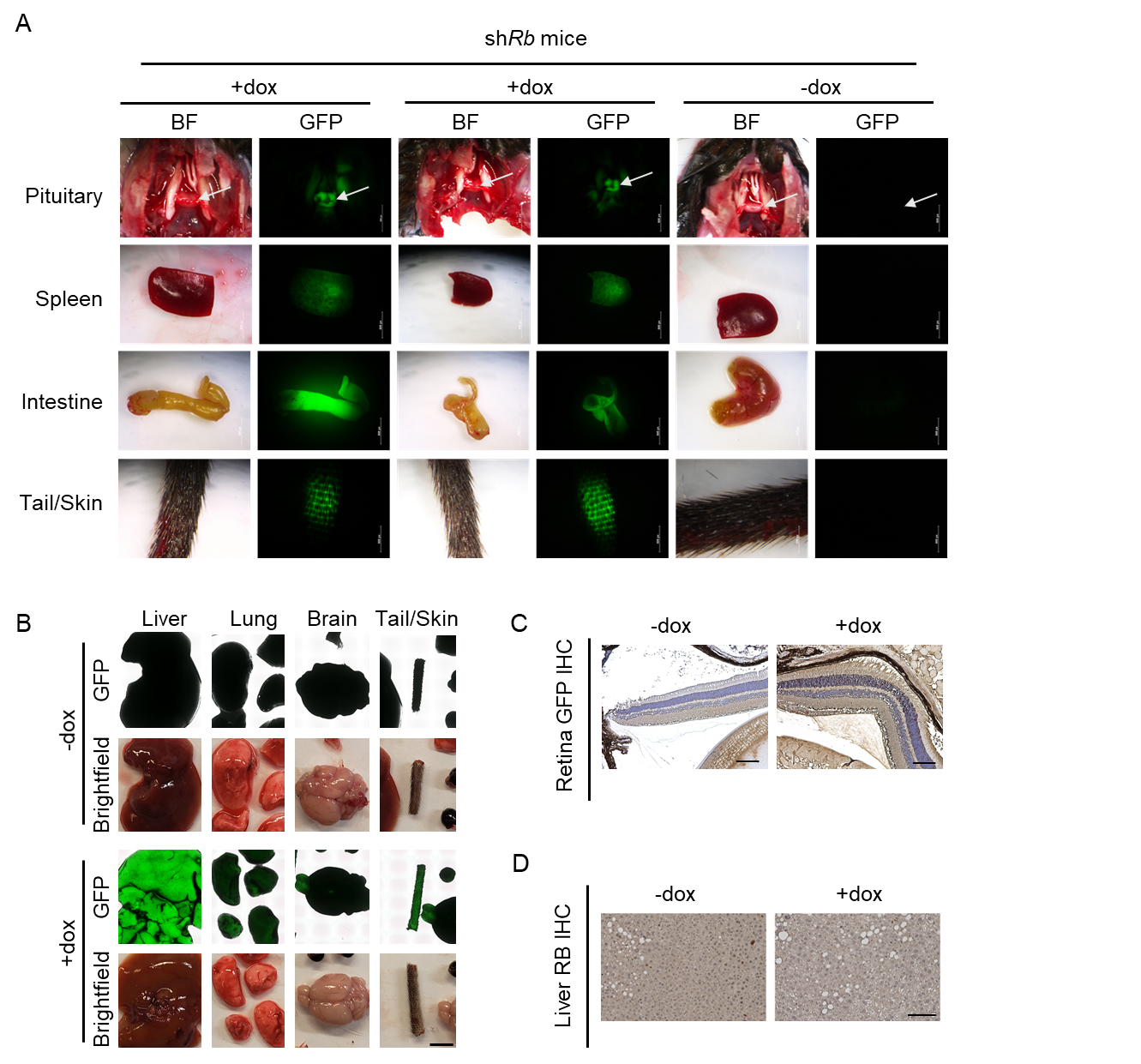

Supplement: S2 Fig — A. Mice were analyzed after 10 days of doxycycline (dox) treatment and GFP expression was detected by fluorescence. Representative brightfield (BF) and fluorescence (GFP) images are shown for two shRb mice +dox. An shRb mouse without dox treatment (-dox) was used as a control. White arrows point to the pituitary gland. Scale bar, 4mm. B. Mice were analyzed after 21 days +dox and GFP expression was detected by fluorescence. Representative images are shown for liver, lung, brain and tail tissue. Scale bar, 4mm. C,D. Representative images of immunohistochemistry analysis for GFP in the retina (C) and RB in the liver liver (D) on sections from mice treated for 21 days +dox compared to no treatment. Scale bar, 100μm. (TIF) [file pgen.1009941.s002.tif]

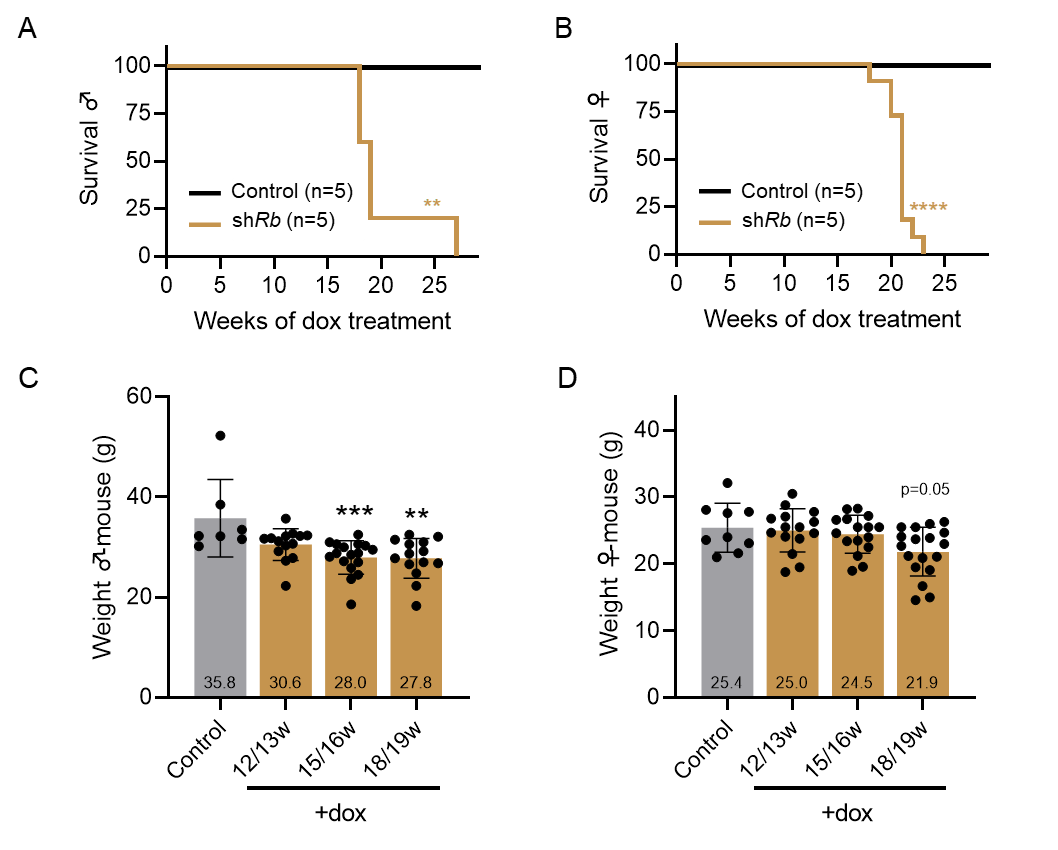

Supplement: S3 Fig — A and B. Survival curves of males (A) and females (B) with Rb knock-down (doxycycline treatment, dox) and in control mice. Significance was calculated using a Log-rank (Mantel-cox) test. C and D. Weight of males (C) and females (D) at different time points (12/13, 15/16, and 18/19 weeks) after Rb knock-down and in control untreated mice (one-way ANOVA against control: **, p<0.01; ***, p<0.01; ****, p<0.0001). (TIF) [file pgen.1009941.s003.tif]

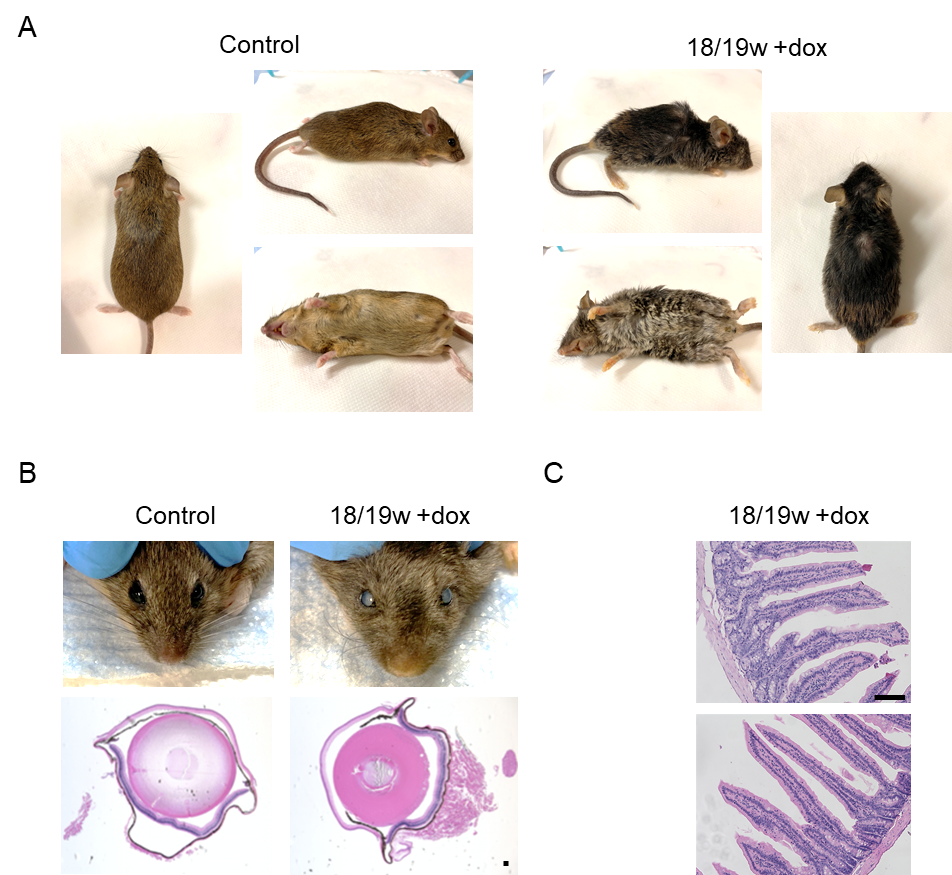

Supplement: S4 Fig — A. Images depicting kyphosis, alopecia, and greying of fur on belly of shRb mice treated with doxycycline (dox) for 18–19 weeks compared to control untreated mice. B. Representative images of eyes in dox treated shRb mice and control mice and H&E (hematoxylin and eosin) staining of eyes from 18w dox treated shRb mice and control mice. Note the intact retina and the lens defects. Scale bar, 100μm. C. Representative images (H&E staining) of small intestine H&E staining from 18w dox treated shRb mice. No gross pathological defects were observed. Scale bar, 100μm. (TIF) [file pgen.1009941.s004.tif]

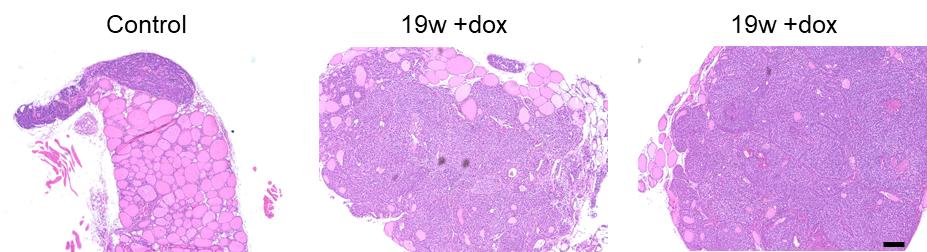

Supplement: S5 Fig — Representative images from H&E (hematoxylin and eosin) stained sections from thyroid glands in control and dox-treated shRb mice (19 weeks). Scale bar, 100μm. (TIF) [file pgen.1009941.s005.tif]

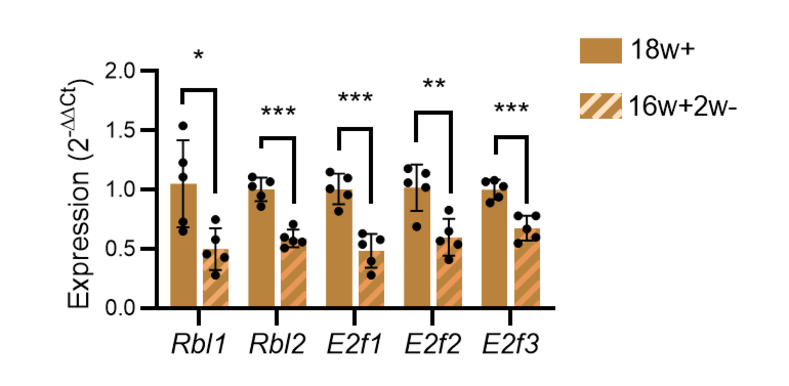

Supplement: S6 Fig — RT-qPCR analysis 2 weeks after RB reintroduction normalized to Rps13. Rbl1 and Rbl2 code for the RB family members p107 and p130, respectively. E2f1/2/3 code for activating E2Fs. Data were analyzed using a two-tailed students t-test; *, p<0.05; **, p<0.01; ***, p<0.001. (TIF) [file pgen.1009941.s006.tif]

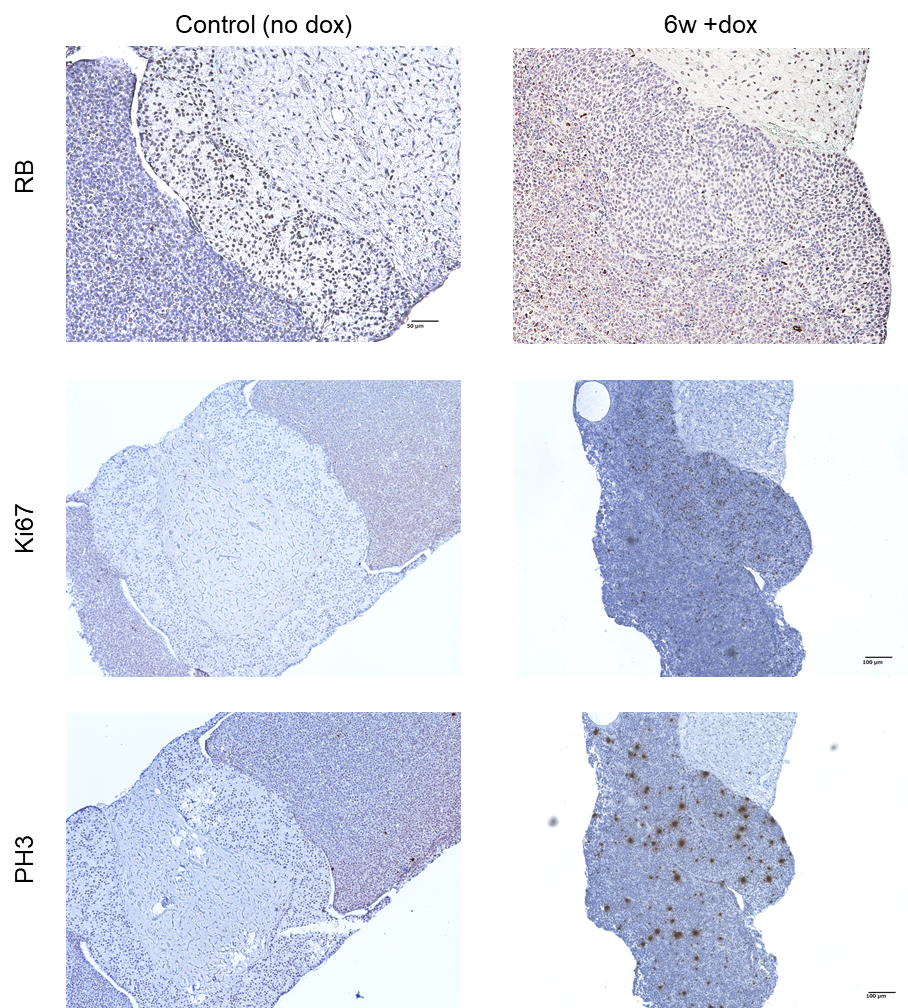

Supplement: S7 Fig — Immunostaining for RB, Ki67, and phospho-histone 3 (PH3) on sections from the pituitary glands of a control mouse (n = 1) and an shRb mouse after 6 weeks of doxycycline (dox) treatment (representative of n = 4 mice). Scale bars, 50μm (RB) and 100μm (Ki67 & PH3). (TIF) [file pgen.1009941.s007.tif]

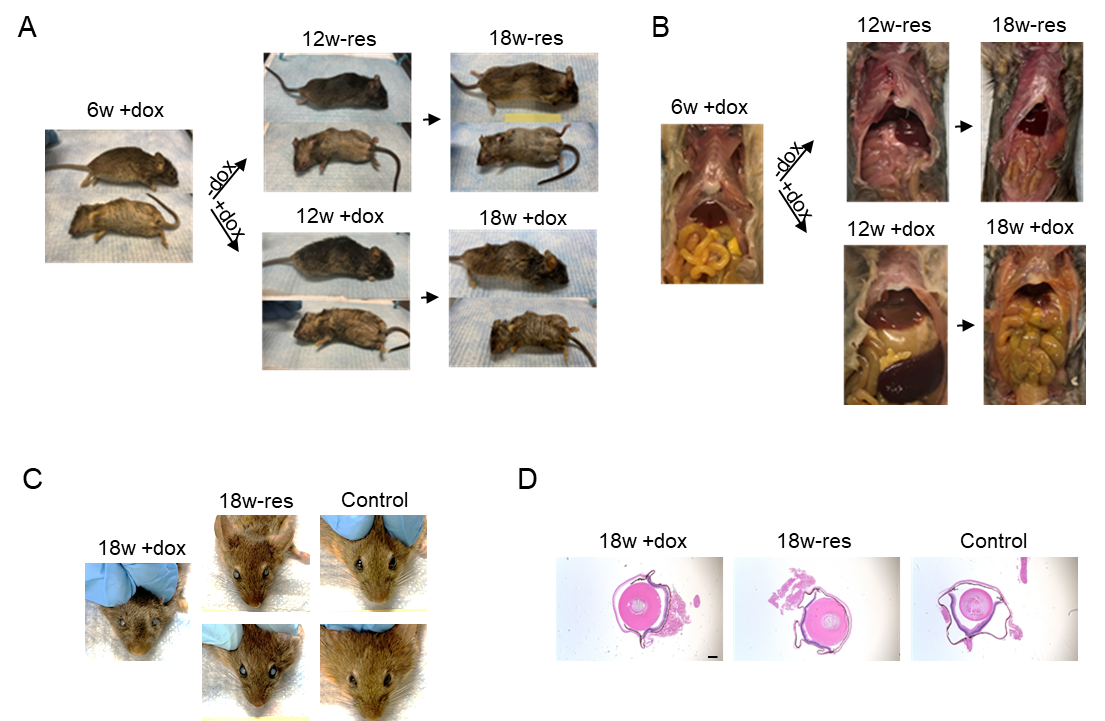

Supplement: S8 Fig — A. Representative images of shRb mice after 6 weeks of doxycycline (6w +dox) treatment and then continued dox treatment or dox removal. B. Representative images of the internal abdominal cavity in mice as in (A). C. Representative images of eyes in different dox treatment groups. D. Representative images of IHC H&E sections of eyes in different dox treatment groups. Scale bar, 400μm. (TIF) [file pgen.1009941.s008.tif]

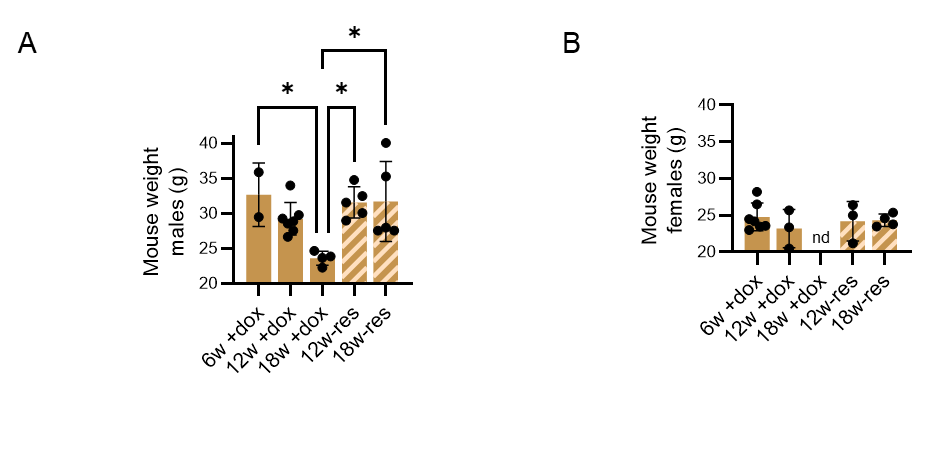

Supplement: S9 Fig — A and B. Weight of males (A) and females (B) at different time points after Rb knock-down (with doxycycline, dox) or re-expression (w, weeks) (one-way ANOVA: *, p<0.05). nd, not determined (no female mice at this time point). (TIF) [file pgen.1009941.s009.tif]

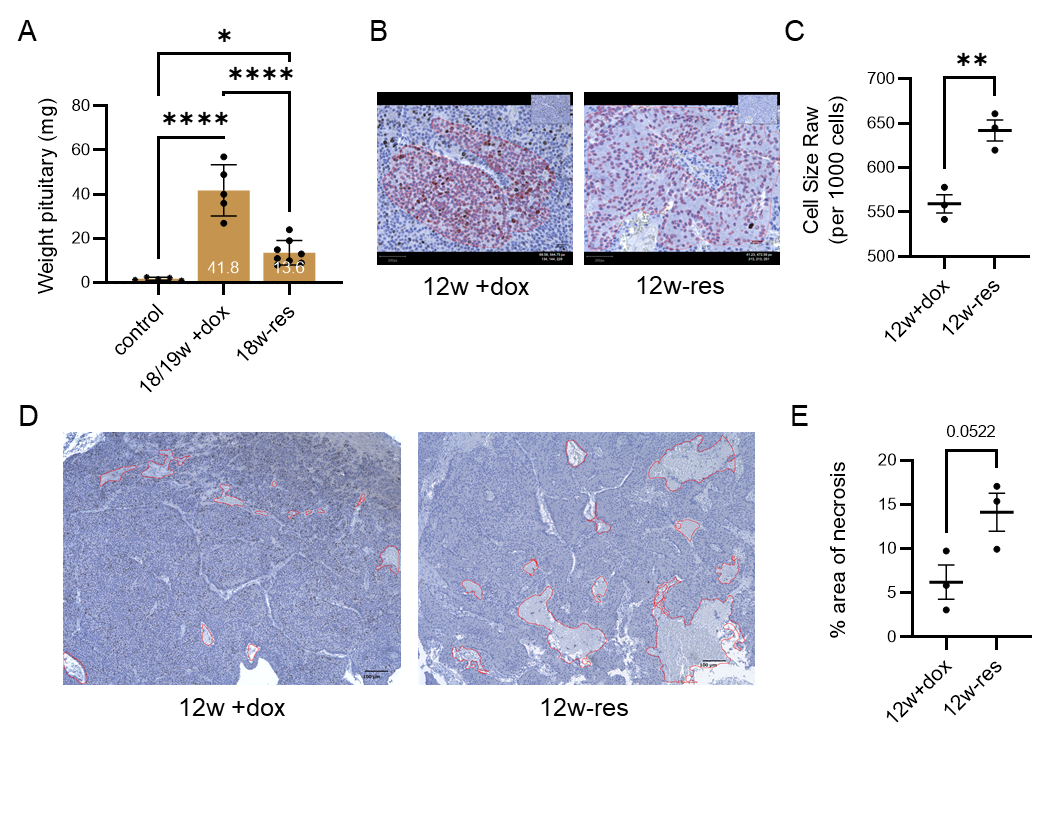

Supplement: S10 Fig — A. Weights of 18w-res pituitary tumors compared to control pituitary glands and 18/19w RB KD tumors (one-way ANOVA analysis: *, p<0.05; **, p<0.01; ****, p<0.0001). B. Representative cell area histograms obtained from Qupath cell detection analysis in computing cell size of 12w+dox (12 weeks on doxycycline) and 12w-res (6 weeks on doxycycline and 6 weeks off) tumor sections. C. Cell size averages (arbitrary units) of tumor sections obtained from histogram analysis in (B) (unpaired t test; *, p<0.05; **, p<0.01). D. Representative images of Hematoxylin-stained sections used to mark areas of necrosis in tumors using the Qupath wand tool. Scale bar, 100μm. E. Computed % area of necrosis in tumor samples from (D) in 12w+dox and 12-res sections (unpaired t test). (TIF) [file pgen.1009941.s010.tif]

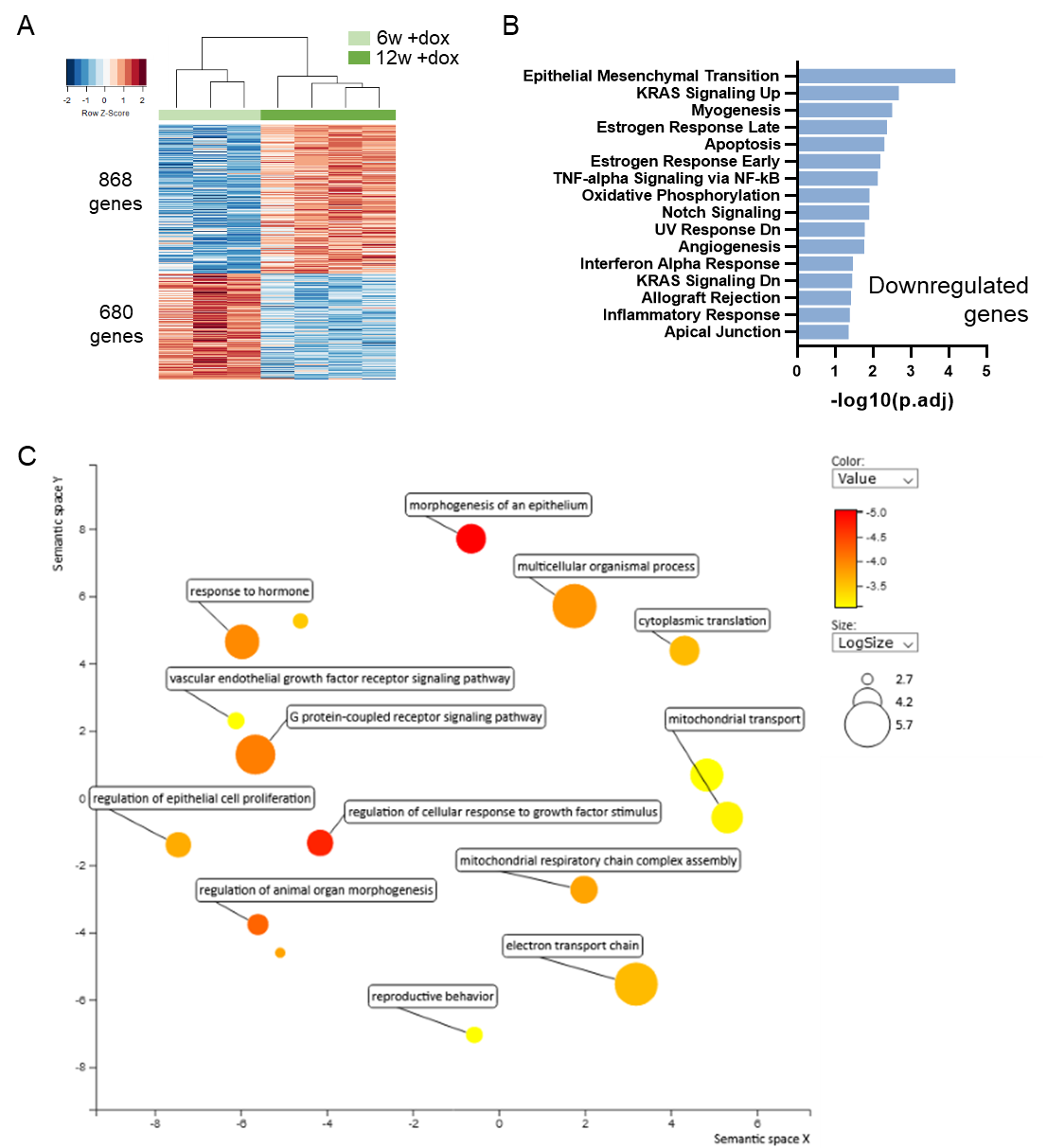

Supplement: S11 Fig — A. Heatmap of differentially-expressed (DE) genes by RNA-seq analysis (DEseq2: log2FC >1; p.adj.<0.05) in pituitary lesions from shRb mice after 6 weeks or 12 weeks in doxycycline (dox) treatment. B. MSigDB Hallmark 2020 enrichment of downregulated genes during tumor progression from the RNA-seq as in (A). Shown are Hallmarks with p.adj.<0.05, analyzed with Enrichr (Kuleshov et al. [58]). Upregulated genes are not enriched for any of the Hallmark datasets. C. Summary of enriched GO Processes in downregulated genes during tumor progression from the RNA-seq as in (A), using Revigo (Supek et al. [60]). Upregulated genes were only enriched for transmembrane transport. (TIF) [file pgen.1009941.s011.tif]

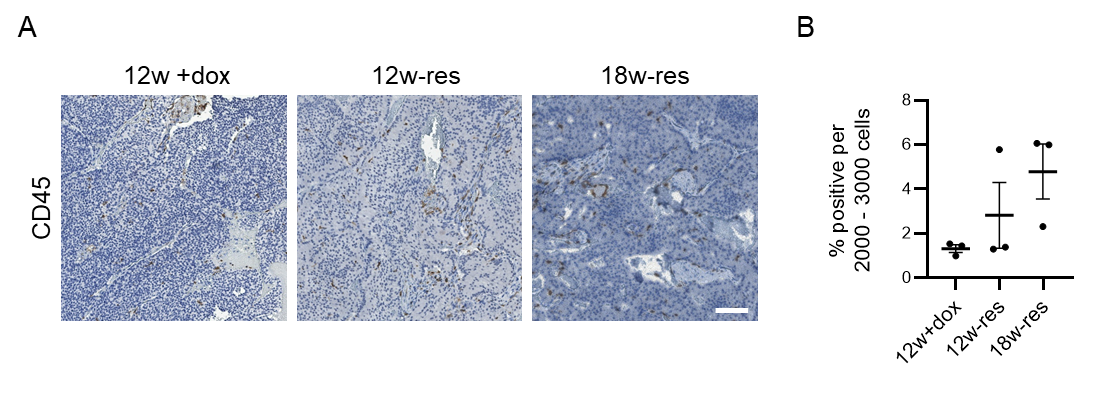

Supplement: S12 Fig — A. Representative images of CD45 staining on sections from different treatment groups. Scale bar, 100μm. B. Quantification of % positive CD45 cells on samples of groups obtained in (A) (unpaired t-test was performed, no significance noted). (TIF) [file pgen.1009941.s012.tif]

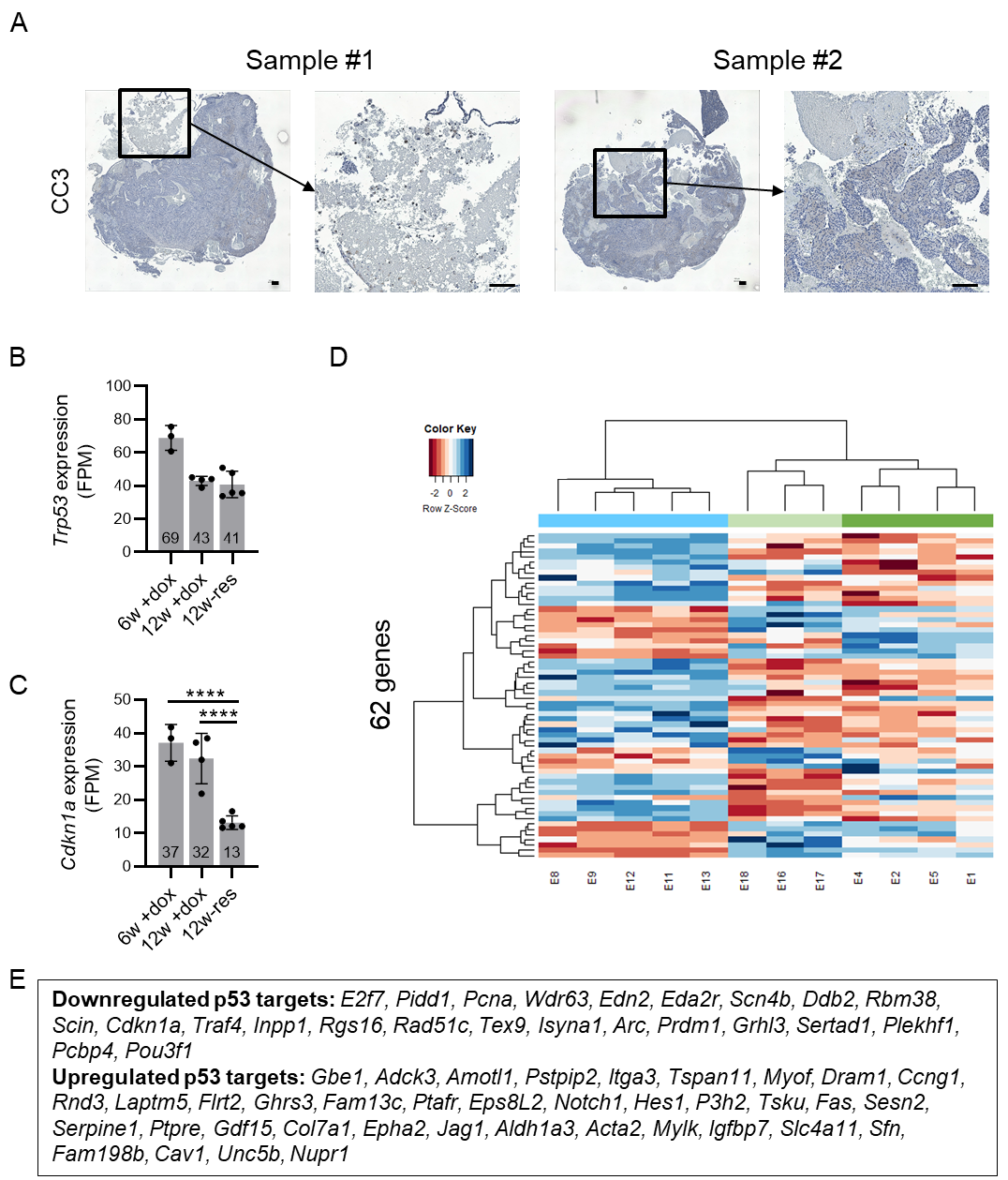

Supplement: S13 Fig — A. Immunostaining for the apoptosis marker cleaved caspase 3 (CC3) on two 12w-res tumor sections (counterstained with hematoxylin). Scale bar, 100μm. B,C. Trp53 (B) and Cdkn1a (C) expression levels from RNA-seq data (****; DEseq2: padj<0.0001, log2FC>|1|). D. Heatmap of RNA-seq results from differentially expressed consensus p53 targets as defined by Fischer et al. (2017) [61]. E. List of differentially-expressed genes from Heatmap in (D). (TIF) [file pgen.1009941.s013.tif]
