# Supplementary material for: RB depletion is required for the continuous growth of tumors initiated by loss of RB
Source: PLoS Genet. 2021 Dec 8;17(12):e1009941. doi: 10.1371/journal.pgen.1009941 (PMC8654178; doi:10.1371/journal.pgen.1009941)
Supplement: Attachment — Submitted filename: 116362_1_rebuttal_2125528_qw9cft.docx [file pgen.1009941.s021.docx]

**Manuscript number:** RC-2021-00805R

**Corresponding author(s):** Julien, Sage

## 1. General Statements

*We thank the Reviewers for their positive assessment of our work. The points raised were minor, in our opinion, and we believe we can address all these points within the next few weeks.*

## 2. Description of the planned revisions

*We provide in the next pages a point-by-point response to the four Reviewers and underlined how we specifically plan to address the points raised. A number of these points will be minor changes to the text, along with new histopathological and immunohistochemistry analyses.*

## 3. Description of the revisions that have already been incorporated in the transferred manuscript

*We have not yet revised the text or carried out and included any new experiments in the transferred manuscript.*

## 4. Description of analyses that authors prefer not to carry out

*One Reviewer suggested that we should perform experiments in mouse embryos and two other Reviewers suggested in the cross-commenting section that such experiments may not be necessary. We provide a statement why we prefer not to carry out these experiments in embryos in the point-by-point response below (based on the results of a pilot study).*

**Reviewer #1 (Evidence, reproducibility and clarity (Required)):**

The manuscript by Alex Doan, Julia Arand et al., describes a novel DOX-inducible shRNA system to silence and re-express Rb ad voluntatem. The analysis is exceptionally well performed, the results of great interest and the potential for follow-up/new discoveries using this model is obvious. The new model allows knockdown of Rb to 20% (on average) of control levels. It is thus less efficient than another model of Rb inactivation/reactivation with floxed Rb alleles (reference 18 and 26), which completely deletes Rb, thus mimicking genetic loss of Rb in cancer - but it may better mimic post-translation inactivation (phosphorylation) of the pRB protein, which incompletely suppresses Rb function. The simplicity and flexibility of the model may also be an asset.

The authors showed that silencing of Rb via shRNA (shRb) induces endocrine ( pituitary and thyroid ) tumors like those observed in Rb+/- mice, and that re-expression of Rb in pituitary tumors (by DOX removal) suppresses cell proliferation, thus demonstrating "addiction" to the continuous loss of this tumor suppressor. Using tissue specific -rtTA - these new shRb mice can be used to test for addiction to Rb loss in tumors of interest such as retinoblastoma (together with p107/p130 loss) or epithelial cancers (together with p53 loss etc). Alternatively, shRb mice may be crossed with a tissue specific floxed p107 or p130, such as Pax6 alpha-enhancer to induce retinoblastoma, or with a floxed p53 with an appropriate Cre line to induce other tumor types in which RB loss promotes cancer and metastasis. It would also be important to compare the two models (shRb vs Ref 26) side by side. However, such additional analysis may be part of a future study; the suggestions below are chiefly on the present work.

We thank the Reviewer for their enthusiasm. We also have a number of ideas for future experiments and have started crosses to investigate lung cancer.

**Major issues**

1. While the authors show that shRb mice develop pituitary and thyroid tumors, a major phenotype of Rb+/- mice, they didn't test for the effect Rb knockdown at earlier stages and whether that would induce embryonic death at ~E13.5 as seen in Rb-/- embryos or 17.5 following placental rescue. This may be technically challenging but should be achieved by - for example - supplementing the drinking water of mating or "plugged" females with DOX. Such analysis is important as it will demonstrate the extent of Rb loss. Indeed, as the authors indicated - tumors may be initiated by rare cells with strong depletion of Rb while the average level may not be sufficiently low in most cells - in which case shRb embryos may survive with interesting phenotypes.

We agree with the Reviewer that this is an area of investigation worth pursuing. We had actually begun to investigate how to knock-down RB during embryonic development but decided not to pursue these experiments at the moment. This decision was based on a pilot experiment where we began doxycycline treatment one week after plug formation in one female: this female gave birth to a viable litter, suggesting that, in this setting, RB levels may not be knocked-down enough to observe the lethal phenotypes observed with full RB knockout during embryogenesis. We note that *Rb^+/-^* embryos develop just fine, so possibly a 70-80% knock-down may not be enough for post-birth lethality in this context. Pups in the litter that was born from that one experiment displayed a variety of phenotypes such as closed eyelids, a wavy fur coat, and impaired mobility, suggesting that RB knock-down in sh*Rb* embryos is sufficient to generate phenotypes. However, these phenotypes were complex and their basis was not readily clear. We hope that the Reviewer will agree with the other Reviewers that the analysis of the consequences of RB knock-down in embryos using sh*Rb* mice should be the scope of future studies.

In this regard, it would also be interesting to quantify Rb expression in DOX-induced mice at a single-cell resolution to gain a better insight as to the variability in Rb depletion in different cells/tissues.

This is a really interesting idea that could help better characterize mice with inducible shRNA molecules. We thought about doing single-cell RNA-sequencing but it is likely that measuring RNA levels may not accurately reflect RB protein levels (because of post-transcriptional regulation of RB mRNA levels by siRNAs, and the long half-life of the RB protein). Detecting mouse RB protein levels is notoriously difficult in the field but we have performed some immunohistochemistry experiments to visualize RB in tissue sections from mice, specifically in liver and pituitary samples. Thus far, we have found that these assays are not always very reliable, especially in terms of being able to quantify and compare expression levels across different sections. To address this point, we will collect more mice and attempt new immunohistochemistry analyses for RB on several mouse tissues in sh*Rb* and control mice, including liver, spleen, intestine, and lung.

2. The authors show that re-expression of Rb by DOX-removal suppresses ki67/proliferation in preformed pituitary tumors. However, the statement on p9 lines 212-213 indicates that tumors in -res mice had no apparent difference in size compared to tumors in mice kept on DOX.

a. How can this be explained? Would a longer follow-up period comparing these cohorts (Dox-removal vs Dox-continuous) uncover differences in tumor progression/size?

The Reviewer raises a good point, and we have been a little bit puzzled by this observation too. Our current explanation is two-fold: (1) possibly the cells become larger when they arrest in G0/G1 (leading to tumors occupying more volume) and (2) there are larger areas of necrosis. To address the point raised by the Reviewer, we will quantify cell size and necrotic areas in mice upon RB re-introduction in pituitary tumors. We will also quantify more precisely tumor weight upon RB re-introduction.

b. P9 lines 237-239 : p53 /apoptotic pathways are induced and necrotic areas are observed - is this verified by staining for apoptotic markers? - is p21 induced as well? - is this consistent with lack of regression?

To address the point raised by the Reviewer, we will check levels of expression of specific p53 targets in the RNA-seq analysis. We will also perform staining for apoptotic markers such as cleaved caspase 3 (CC3) on sections from pituitary tumors.

c. One would expect that some tumor cells will fail to re-express Rb upon Dox removal and form clones that escape Rb-reactivation and continue to grow. Do the authors see some islands of Rb-negative - ki67+ cells in the 12s-res, a18w-res lesions or after a longer period off DOX?

There are reports of transgenes being silenced, which may then lead to the situation described by the Reviewer. We re-examined more carefully the section we had stained for Ki67 and RB and have found no evidence of “islands” of cells in which RB was not re-expressed or Ki67 expression not silenced. We will mention this point in the text of the revised manuscript.

**Minor issues**

p5 line 101 - It would be helpful to state the level of knockdown (80%) in the text.

We will revise the text to mention the level of knock-down.

p5 lines 116-118 - no expression in lung/brain: can this be shown? Where exactly? What about expression in retina?

To address the point raised by the reviewer, we are generating new sh*Rb* and control mice and will take images of GFP expression in the lung, brain, and eye, in addition to the previous tissues we provided images for in the initial supplemental figure. Our pilot analysis suggests that, compared to the liver, intestine, and small intestine, there is minimal GFP expression in the new tissues analyzed. Weak expression seems dispersed throughout the lung lobes, while concentrated weak signal seems present in the spinal cord region of the brain and not in the bulk region of the brain itself. We also see dispersed weak GFP expression in the eye. These new findings, which will be repeated before submission of the revised version, support our initial observation that the sh*Rb* system may not be expressed strongly in these tissues.

In addition, the authors state that low expression in the lung is due to low level expression of the ROSA locus that drives the M2rtTA in this tissue. But a lung-specific-rtTA may efficiently induce shRNA-Rb in the lung - allowing analysis of Rb loss in lung cancer and direct comparison (in future studies) with Ref. 18.

We agree with the Reviewer. As mentioned above, we are interested in lung cancer and this would be one way to bypass the low expression from the *Rosa26* promoter in the lungs.

Is low delivery of DOX to the brain due to the blood brain barrier (BBB)? Can this be resolved? These two issues may be briefly discussed.

We will discuss this issue and will provide references in the revised version of the manuscript.

Fig. 5B - arrows pointing at tumors should be added.

We will add arrows to the figure in the revised version of the manuscript.

P9 235. "a normal cell cycle" - what's "normal"? Define/ rephrase.

P10 267 "including compared" - rephrase.

Fig S7 title "phenotypes of shRb mice UP RB -re-expression" - change UP to AFTER?

We will rephrase these sentences in the revised manuscript.

Fig S4B - what happens to the lens/cataract following re-expression of Rb?

This is an interesting question. We would not expect these defects to disappear upon RB re-expression because the lens does not regenerate naturally in adult mice but we have not specifically examined this phenotype in the rescue animals. To address the point raised by the Reviewer, we will provide images for the lens of mice after RB re-introduction.

Reviewer #1 (Significance (Required)):

See above.

**Reviewer #2 (Evidence, reproducibility and clarity (Required)):**

**Summary:**

Doan and colleagues present here a new mouse model which expresses a short hairpin against the RB tumor suppressor from a doxycycline-inducible promoter in the Col1a1 locus. Using this model, they demonstrate that in vivo knockdown causes defects in multiple organs. These defects included tumor growth in pituitaries and thyroids, which is also seen in Rb+/- mice or chimeric Rb-/- mice. They then provide evidence that re-establishment of RB expression by withdrawing the doxycycline treatment again blocks proliferation of the pituitary tumors.

**Major comments:**

Overall, this is a very well-written paper. The results look robust and reproducible, and in most experiments, the appropriate controls were included. The conclusions are supported by the data and the authors are not overselling their data. However ,the analysis of the mouse model could have been done in more detail to better characterize and understand the lesions induced by knocking down and re-expressing Rb.

We thank the Reviewer for this fair assessment of our work. Our goal was to describe this new model rigorously. Future experiments using sh*Rb* mice in different organs and contexts will be critical to better understand the molecular and cellular consequences of RB loss and re-introduction *in vivo*.

*Specific points:**

- In figure 2 the authors describe multiple phenotypes seen after doxycycline treatment. These include ageing-like phenotypes (kyphosis, greying fur) and enlargement of intestinal organs and inflammation. Are these phenotypes consistent with other mouse models of RB loss? What are the underlying mechanisms? Could off-target effects of the shRB construct (partly) explain those phenotypes? –

The Reviewer is raising an interesting point. Overall, the phenotypes described in the sh*Rb*  model are very similar to what has been observed or described in *Rb* knockout mice, although for some of these phenotypes, previous studies did not necessarily provide a detailed description. To address the point raised by the Reviewer, we will provide a comparison between the phenotypes described in our study and phenotypes described for *Rb* mutant mice in previous studies.

Following up on this: when I looked at the "organ enlargements" described in Figure 2D it seems that the intestines of the shRB mice are overfilled with food. Do these mice have problems with intestinal absorption and gut motility? Previous studies suggested that RB is important for differentiation of intestinal epithelium (Chong et al. Nature 2009); does this play a role here? Microscopic analysis of the intestines would be required to at least provide some basic insight. Could the intestinal lesions explain the weight loss observed in mice where Rb was knocked down?

Our first thought was that these phenotypes were simply a sign that sh*Rb* mice get sick with time but the Reviewer brings up a good point. To address this point, we will provide histology images from control and mutant intestines.

The authors provide clear arguments why they chose to knock down RB for only 6 weeks in the rescue experiments. But even though they show increased Ki67 staining at 6 weeks, it's not clear whether at that stage the pituitaries are still preneoplastic, or already have developed a pituitary neoplasm. It would be important to know the status of the pituitary tissue at 6 weeks through histological analysis performed by an experienced pathologist.

It would be misleading to characterize the pituitary lesions at these early time points as adenocarcinomas but previous studies call them adenomas. To address the point raised by the Reviewer, we will work with Dr. Hannes Vogel, an experienced pathologist, to provide a description of these early pituitary gland lesions.

Following up on this: did the rescue experiment show tumor regression? Pituitary masses are shown in the RB knockdown experiment (Figure 4) but not in the rescue experiment (Figure 5). It is only briefly stated that there is no difference, referring to Figure 5B, but these macroscopic photos are very hard to judge, and may not representative; hence a proper quantification of pituitary weights and histological analysis must be shown. Furthermore, what is the degree of necrosis/apoptosis/ senescence for the experiments outline in figure 5.

- Related to this above point, it is surprising that the pituitary tumors are not smaller in the 18w-res mice than in the dox-treated condition. The authors should discuss the possible reasons.

The Reviewer is raising a similar point as Reviewer #1 (point #2). We have been a little bit puzzled by this observation too. Our current explanation is two-fold: (1) possibly the cells become larger when they arrest in G0/G1 (leading to tumors occupying more volume) and (2) there are larger areas of necrosis. To address the point raised by the Reviewer, we will check levels of expression of specific p53 targets in the RNA-seq analysis. We will also perform staining for apoptotic markers such as cleaved caspase 3 (CC3) on sections from pituitary tumors. In addition, we will quantify cell size and necrotic areas in mice upon RB re-introduction in pituitary tumors. We will also quantify more precisely tumor weight upon RB re-introduction.

**Minor comments:**

-Line 229: "... variation along PCA while PC2...". It looks like the authors mean PC1 instead of PCA here.

We will correct this mistake in the revised manuscript.

-Figure 5H: the authors conclude that the variation along PC1 is mostly caused by RB re-expression, while PC2 variation is caused by tumor progression. If that is true, why did the rescue experiment only partly inhibit tumor progression? Have the authors analyzed the PCA loadings to evaluate which specific (groups of) genes drive variation in PC1 and PC2 respectively? A more in-depth analysis could help gaining better mechanistic insight into the observed phenotypes.

The Reviewer brings up good point. As discussed above, partial tumor inhibition measured by tumor area may be misleading (larger cells, more necrosis) and the Ki67 levels, which become barely detectable, may be a better marker of how potent RB re-expression is to suppress the growth of these pituitary tumors. To address the point raised by the Reviewer regarding the drivers of the principal component analysis (PCA), we will perform additional computational analyses of the RNA-seq data.

Reviewer #2 (Significance (Required)):

The authors present here a new mouse model in which RB can be reversibly depleted using a doxycycline-inducible shRNA. This allowed the authors to study investigate if re-expression of RB loss-induced tumors. Interestingly the proliferation of pituitary tumor cells strongly depends on the RB status. In a previous study, others already showed the effect of re-expressing RB in KRAS/P53/Rb mutant mouse lung adenocarcinoma [1]. The results of the present study seemingly contradict that previous work, because RB expression did not halt cancer cell proliferation in lung adenocarcinomas but in pituitary is does. However, the models are different and potential tissue specific function for RB and the different genetic context could explain the observations. Probably the tumor stage at the moment of re-expression is of critical importance as well, but unfortunately the authors have not address this in the presented model.

This is a really good point raised by the Reviewer. We will make sure in the revised discussion to emphasize the context-dependency of the requirements for both RB loss in cancer initiation and cancer maintenance. The idea of re-expressing RB at different times during tumor development is also what we had originally in mind and what we actually did and presented in this manuscript. Unfortunately, when we re-expressed RB in late-stage tumors, we observed cell cycle arrest but were not able to study these mice for a long time because of their death from these tumors. This is why we also performed experiments with earlier-stage tumors in the last part of the manuscript. A limitation of the sh*Rb* model is that it takes 2-3 weeks to re-express RB, which limits how acutely these re-introduction experiments can be performed. It will be interesting in the future to use the sh*Rb* mice in lung cancer models and compare with the work from the Feldser lab using their XTR allele (Walter et al., 2019 – PMID: 31043741).

This study is of interest to the field of cancer genetics, and especially to those interested in understanding the molecular actions of tumor suppressor proteins, including RB/E2F. For researchers working with genetic mouse models, the approaches in this study could be of interest, although the approach is not entirely new. For example, APC knockdown and re-expression has been studied in a similar model [2].

Dr. Dow is a co-author on our study and was the lead author in the APC study (Dow et al., 2015 – PMID: 26091037). It is true that the use of inducible shRNA in transgenic mice is not novel. We believe that it is still important to develop and characterize different models for different tumor suppressors. These different models may provide novel insights into the different mechanisms of action of tumor suppressors in different cancer types. We will revise the text to discuss these points.

It is a bit difficult to see how these results could be translated to human cancer patients. And although of interest and well-performed, this study does not provide a completely new concept or idea in my view, since it expected that re-expression of Rb will stop cell proliferation.

In terms of human cancer relevance, we note that human pituitary tumors show silencing of *RB1* by DNA methylation (30-35% of cases) as well as *CDKN2A* coding for p16^INK4A^ (60-70% of cases) (Yoshino et al., 2007 – PMID: 1721655; Ogino et al., 2005 – PMID: 15892297; Simpson et al., 2000; – PMID: 10728677)., indicating that pituitary tumors in RB-deficient mice are a relevant model for this human cancer type. We also want to emphasize that a main goal for developing sh*Rb* mice is to discover key mechanisms of action of RB. Studies in mouse pituitary and thyroid tumors are relevant to other cancer types, as shown before. For example, the demonstration that E2F is a key mediator of cancer phenotypes downstream of loss of RB was first made in mouse pituitary tumors (Yamasaki et al., 1998 – PMID: 9537419). That said, we fully agree with the Reviewer that our studies will be even more relevant in the context of other cancer types, including lung and breast cancer. We hope that the Reviewer will agree with us that these studies belong to future manuscripts.

It was also expected in the Feldser study (Walter et al., 2019 – PMID: 31043741) that once cells have arrested upon RB re-introduction, they would remain arrested. However, cancer cells with RB re-introduction only stopped cycling transiently, so one could argue that our “expected” observation may not have been 100% expected and that it is important to test this model in several cancer contexts.

We will make sure in the revised text to discuss these points more thoroughly.

My field of expertise is cell cycle control, E2F/RB, cell biology, transcriptomics, and genetic mouse cancer models.

[1] Walter DM, Yates TJ, Ruiz-Torres M, Kim-Kiselak C, Gudiel AA, Deshpande C, et al. RB constrains lineage fidelity and multiple stages of tumour progression and metastasis. Nature. 2019;569(7756):423-7. doi: 10.1038/s41586-019-1172-9.s

[2] Dow LE, O'Rourke KP, Simon J, Tschaharganeh DF, van Es JH, Clevers H, et al. Apc Restoration Promotes Cellular Differentiation and Reestablishes Crypt Homeostasis in Colorectal Cancer. Cell. 2015;161(7):1539-52. Epub 2015/06/20. doi:649 10.1016/j.cell.2015.05.033.

**Referee Cross-commenting**

I agree also with the other reviewer comments. Similar to reviewer 3 I believe that performing additional developmental studies are beyond the scope of this study. Furthermore, studying RB function during development is complex because you have to take into account both placental and embryonic specific function of RB. These studies would take at least another 2 years to perform.

We had actually begun to investigate how to knock-down RB during embryonic development, as discussed in response to the comment from Reviewer #1 but decided not to pursue these experiments because the complex phenotypes observed in pilot experiments indicate that such developmental studies will require various experimental approaches and assays.

**Reviewer #3 (Evidence, reproducibility and clarity (Required)):**

This manuscript uses a newly developed inducible shRNA mouse model to study the effects of reversible RB depletion and reactivation in a broad array of tissues. The authors find that RB depletion induces several phenotypes associated with aging and inflammation. Similar to Rb knockout mice, mice expressing the inducible shRb transgene also develop pituitary and thyroid tumors. This result confirms the accuracy of this model for studying the impact of RB loss. An advantage of this model, however, is that RB can be reactivated to study how this impacts phenotypes, including tumor progression. The authors find that re-expression of RB results in a stabilization of phenotypes, induces a sustained inhibition of cell proliferation in pituitary adenomas, and prevents thyroid tumorigenesis. The findings are convincing and well controlled.

Based on these findings the authors conclude that continuous RB loss is required for the maintenance of tumor phenotypes initiated by loss of RB. This conclusion is in contrast to a previously reported study using a genetic recombination mouse model in which Rb re-expression resulted in only a transient inhibition of cancer cell proliferation in a K-ras mediated lung tumors due to upregulation of CDK2 activity. Thus, the above conclusion is valid if considering tumors "initiated by loss of RB" but this is not necessarily the case for all tumor types involving loss of RB function. This context-dependent effect of RB reactivation should be further discussed, particularly in light of understanding which cancers might respond to CDK inhibition and which might quickly develop resistance. Otherwise the methods are presented clearly and experiments are adequately replicated.

We fully agree with these comments from the Reviewer. As discussed above in response to the comment from Reviewer #2, we will make sure in the revised discussion to emphasize the context-dependency of the requirements for both RB loss in cancer initiation and cancer maintenance.

**Some minor comments.**

1) In the manuscript text, line 104, the authors state that the shRb5-4 mESC clone was used to generate transgenic mice because, in part, it had a normal karyotype. However, Figure S1D indicates that clone 4 is aneuploid because it is marked with a star (clone 4*). Doesn't shRb5-4 indicate the shRb5 transgene, clone 4? Please explain/address this discrepancy.

We apologized for this mistake, we meant to write “euploid” for clones 2 and 4. We will correct this mistake in the revised version of the manuscript.

2) The phenotype description of the shRb expressing mice on line 134 is unclear. Do you mean 4 out of 4 mice expressing shRb had a scruffy greying hair coat and....

We thank the Reviewer for picking this other mistake, We will rewrite this sentence, 4/4 sh*Rb* mice showed these phenotypes.

3) Can the authors speculate as to why loss of RB results in phenotypes associated with aging? Have they looked at senescence in affected tissues?

We were struck when we analyzed the phenotypes of these mice carefully how they resemble some aging phenotypes. It is very likely that some (or most) of these phenotypes are due to hormonal imbalances (with tumors growing in the pituitary and the thyroid glands). In support of this idea, similar phenotypes have been described (although not in great detail) in *Rb^+/-^* mice and mice in which *Rb* was deleted specifically in the pituitary gland (Vooijs et al., 1998 – PMID: 9671308). We did not examine senescence in tissues and organs in these mice and hope that the Reviewer will agree with us that studies of cell autonomous and non-cell autonomous of organismal and cellular aging are beyond the scope of this first description of sh*Rb* mice. This point is similar to the first point raised by Reviewer #2 and we will provide a comparison between the phenotypes described in our study and phenotypes described for *Rb* mutant mice in previous studies to highlight some of these phenotypes, especially those related to accelerated aging.

Reviewer #3 (Significance (Required)):

The significance of this work includes the development of a new mouse model of RB deficiency that complements existing Rb mouse models. An advantage of this new mouse model is the ability reactivate RB to study how therapeutic interventions that attempt to reactivate RB, such as CDK inhibitors, impact cancer progression long term. The authors show that reactivation of RB in at least some contexts can have long-term benefits in preventing cancer progression. This adds to the existing literature that reactivation of RB using various strategies can be an effective therapeutic approach in some, but not all settings. This model will be useful in the future in helping to define the contexts in which RB reactivation is beneficial. As a researcher in the E2F-RB field that uses mouse models, I found this study elegant and well done despite having to overcome some technical issues, such as the slow reactivation of RB.

**Referee Cross-commenting**

I agree with the other reviewer's comments although I think asking the authors to perform the embryonic development studies may be asking too much. We all seem to agree it is a solid study.

We thank the Reviewer for this positive view of our work. As discussed above, we are interested in the possible use of sh*Rb* mice in mouse embryos but this may be the focus of a different manuscript because the complex phenotypes observed in pilot experiments indicate that such developmental studies will require various experimental approaches and assays.

**Reviewer #4 (Evidence, reproducibility and clarity (Required)):**

The retinoblastoma tumor suppressor protein, RB, plays a major role in constraining both cancer development and cancer progression in multiple tissue types. Despite clinical observations supporting the concept that RB loss promotes tumor phenotypes, significant gaps remain in discerning the overarching impact of RB status as a function of tumor progression. The present study describes a new mouse model, generated toward the goal of understanding the impact of RB loss and subsequent reactivation in vivo. Key findings of the study demonstrate that the new regulatable model systems develop the expected tumor profile after RB loss (eg thyroid and pituitary tumors), and that subsequent RB re-expression induces cell cycle arrest in pituitary adenomas. The collective observations provide compelling evidence that RB depletion is required for the continuous growth of tumors initiated by RB loss, thus providing new impetus for developing strategies beyond CDK4/ inhibitors to re-engage RB pathway function.

**Critique:**

This is a well conducted study that reports generation of an elegant new model system for studying RB function in vivo. The data shown generally support the stated conclusions, and the study will be of significant impact for the cell cycle and tumor suppressor field. Addressing the following minor concerns would further increase the impact of the findings.

We thank the Reviewer for this positive view of our work.

1. It would be of benefit to determine the impact of RB depletion and reintroduction on potential compensatory pathways (e.g., p107 and p130 expression), and to report the impact on individual activator E2F family members.

This is a good point raised by the Reviewer, as functional overlap and compensation are well known to exist between RB family members. Over the years, we have not been successful in establishing reliable immunostaining protocols for p107 and p130 unfortunately. To address the point raised by the Reviewer, we will perform RT-qPCR for *p107* (*Rbl1*) and *p130* (*Rbl2*) and *E2f* genes in pituitary tumor samples following RB re-introduction.

2. The interferon response pathway appears to be significantly altered as a function of RB status (Figure 5). It would be of interest to determine or speculate the impact of this finding as related to observed tumor responses.

There is emerging evidence that the RB pathway is connected with immune pathways, including the interferon pathway (see Knudsen et al., 2021, as a recent example – PMID: 32424005). We will make sure to include the proper references in the revised manuscript. We were initially intrigued by this observation in our RNA-seq and wondered whether re-introduction of RB may affect the microenvironment of pituitary tumors, especially in regard to immune cells. Our first analysis of the RNA-seq data using CIBERSORT to deconvolute possible signals from immune cells did not reveal any striking difference. To address the point raised by the Reviewer, we will perform immunostaining for immune cells on sections from pituitary tumors after RB re-introduction.

3. In addition to above, the observation of EMT related gene expression changes could be of significant relevance. It will be important to determine whether cell fate changes or markers of plasticity occur as a result of changing RB status.

We and others have connected RB to cell plasticity and cell fate decisions (e.g., Walter et al., 2019 – PMID: 31043741; Kareta et al., 2015 – PMID: 25467916). The comment by the Reviewer encompasses a broad range of cellular and molecular processes and it is difficult for us to think about the best way to characterize these processes in the current study. Thus, to address the point raised by the Reviewer, we revised the Discussion section and mentioned the possible use of this sh*Rb* model in the future to address how RB controls cell fate decisions in various contexts.

Reviewer #4 (Significance (Required)):

see above.
