# Supplementary material for: RB depletion is required for the continuous growth of tumors initiated by loss of RB
Source: PLoS Genet. 2021 Dec 8;17(12):e1009941. doi: 10.1371/journal.pgen.1009941 (PMC8654178; doi:10.1371/journal.pgen.1009941)
Supplement: Attachment — Submitted filename: PGENETICS-D-21-00958 Response to Reviewers.docx [file pgen.1009941.s022.docx]

**Reviewer #1 (Evidence, reproducibility and clarity (Required)):**

The manuscript by Alex Doan, Julia Arand et al., describes a novel DOX-inducible shRNA system to silence and re-express Rb ad voluntatem. The analysis is exceptionally well performed, the results of great interest and the potential for follow-up/new discoveries using this model is obvious. The new model allows knockdown of Rb to 20% (on average) of control levels. It is thus less efficient than another model of Rb inactivation/reactivation with floxed Rb alleles (reference 18 and 26), which completely deletes Rb, thus mimicking genetic loss of Rb in cancer - but it may better mimic post-translation inactivation (phosphorylation) of the pRB protein, which incompletely suppresses Rb function. The simplicity and flexibility of the model may also be an asset.

The authors showed that silencing of Rb via shRNA (shRb) induces endocrine ( pituitary and thyroid ) tumors like those observed in Rb+/- mice, and that re-expression of Rb in pituitary tumors (by DOX removal) suppresses cell proliferation, thus demonstrating "addiction" to the continuous loss of this tumor suppressor. Using tissue specific -rtTA - these new shRb mice can be used to test for addiction to Rb loss in tumors of interest such as retinoblastoma (together with p107/p130 loss) or epithelial cancers (together with p53 loss etc). Alternatively, shRb mice may be crossed with a tissue specific floxed p107 or p130, such as Pax6 alpha-enhancer to induce retinoblastoma, or with a floxed p53 with an appropriate Cre line to induce other tumor types in which RB loss promotes cancer and metastasis. It would also be important to compare the two models (shRb vs Ref 26) side by side. However, such additional analysis may be part of a future study; the suggestions below are chiefly on the present work.

We thank the Reviewer for their enthusiasm. We also have a number of ideas for future experiments and have for example started crosses to investigate lung cancer.

**Major issues**

1. While the authors show that shRb mice develop pituitary and thyroid tumors, a major phenotype of Rb+/- mice, they didn't test for the effect Rb knockdown at earlier stages and whether that would induce embryonic death at ~E13.5 as seen in Rb-/- embryos or 17.5 following placental rescue. This may be technically challenging but should be achieved by - for example - supplementing the drinking water of mating or "plugged" females with DOX. Such analysis is important as it will demonstrate the extent of Rb loss. Indeed, as the authors indicated - tumors may be initiated by rare cells with strong depletion of Rb while the average level may not be sufficiently low in most cells - in which case shRb embryos may survive with interesting phenotypes.

We agree with the Reviewer that this is an area of investigation worth pursuing. We had begun to investigate how to knock-down RB during embryonic development but decided not to pursue these experiments further for this manuscript. This decision was based on a pilot experiment where we began doxycycline treatment one week after plug formation in one female: this female gave birth to a viable litter, suggesting that, in this setting, RB levels may not be knocked-down enough to observe the lethal phenotypes observed with full RB knockout during embryogenesis. We note that *Rb^+/-^* embryos develop just fine, so possibly a 70-80% knock-down (which is what we observed in fibroblasts and ES cells) may not be enough for embryonic lethality in this context. It is also possible that the dose of doxycycline reaching embryos may not be optimal. Pups in the litter that was born from that one experiment displayed a variety of phenotypes such as closed eyelids, a wavy fur coat, and impaired mobility, suggesting that RB knock-down in sh*Rb* embryos is sufficient to generate some phenotypes. However, these phenotypes were complex, and their basis was not readily clear. We hope that the Reviewer will agree with the other Reviewers that the analysis of the consequences of RB knock-down in embryos using sh*Rb* mice should be the scope of future studies. To address this point in the revised manuscript, we included a sentence in the Discussion.

In this regard, it would also be interesting to quantify Rb expression in DOX-induced mice at a single-cell resolution to gain a better insight as to the variability in Rb depletion in different cells/tissues.

In the first version of the manuscript, we had been able to perform some immunohistochemistry experiments to visualize RB in pituitary tumor samples, which showed consistent downregulation of RB across the lesion. To address the point raised by the Reviewer, we improved our immunostaining protocol and performed new immunohistochemistry staining for RB on different mouse tissues in sh*Rb* and control mice. We were not able to reproducibly detect RB on liver sections, which could be due to lower RB levels in this tissue or poorer access to the epitopes recognized by the antibodies. However, we were able to get strong and reproducible staining on sections from the epithelium of the small intestine and the lung bronchiolar epithelium. Nearly all the epithelial cells examined showed significant loss of staining for RB in sh*Rb* mice, showing little variability in the efficiency of the knock-down. We added these observations in **new Fig. 1E-F** and **new Supplementary S2D**.

2. The authors show that re-expression of Rb by DOX-removal suppresses ki67/proliferation in preformed pituitary tumors. However, the statement on p9 lines 212-213 indicates that tumors in -res mice had no apparent difference in size compared to tumors in mice kept on DOX.

a. How can this be explained? Would a longer follow-up period comparing these cohorts (Dox-removal vs Dox-continuous) uncover differences in tumor progression/size?

The Reviewer raises a good point, and we were a little bit puzzled initially by this observation too. To address the point raised by the Reviewer, we quantified more precisely tumor weight upon RB re-introduction. We found again that re-expressing RB is not sufficient to completely prevent an increase in the size of pituitary tumors, but weight increase is lower than in mice kept on doxycycline (**new Supplementary Fig. S10A**). Our current explanation is two-fold: (1) RB re-expression takes a few weeks, and tumor proliferation and growth thus does not stop right away; (2) the cells become larger when they arrest in G0/G1 (leading to tumors occupying more volume) (quantified in **new Supplementary Fig. S10B,C**); and (3) there are larger areas of necrosis (quantified in **new Supplementary Fig. S10D,E**).

b. P9 lines 237-239 : p53 /apoptotic pathways are induced and necrotic areas are observed - is this verified by staining for apoptotic markers? - is p21 induced as well? - is this consistent with lack of regression?

To address the point raised by the Reviewer, we checked levels of expression of specific p53 targets in the RNA-seq analysis. *p53* (*Trp53*) itself shows no change in long-term rescue samples versus time-matched doxycycline-treated samples, and it is downregulated upon tumor progression (**new Supplementary Fig. S13B**). The p53 target *p21* (*Cdkn1a*) is downregulated upon long-term rescue without expression change during tumor progression (**new Supplementary Fig. S13C**). We had a closer look at the expression of consensus p53 targets [1] and found 62 of them to be differentially expressed upon RB rescue (**new Supplementary Fig. S13D**). Downregulated genes (in total 24) are enriched for genes involved in DNA damage response or G1/S transition (e.g., *Pcna*, *E2f7*, *Pidd1*, *Rad51c*) while upregulated genes (in total 32) are enriched for genes involved in regulation of differentiation, response to chemical stress, wound healing, extracellular matrix organization (e.g., *Notch1*, *Hes1*, *Cav1*, *Serpine1*, *Itga3*) (**new Supplementary S13E**). These data suggest that specific programs downstream of p53 may be changing upon RB re-expression.

To further address this point, we performed staining for the apoptotic marker cleaved caspase 3 (CC3) on sections from pituitary tumors. We observed some CC3+ signal in necrotic areas but little-to-no signal within tumors, suggest that apoptosis is not a major consequence of RB re-introduction in this context, at least at the time points examined. We provided representative images of these images in a **new Supplementary Fig. S13A**.

c. One would expect that some tumor cells will fail to re-express Rb upon Dox removal and form clones that escape Rb-reactivation and continue to grow. Do the authors see some islands of Rb-negative - ki67+ cells in the 12s-res, a18w-res lesions or after a longer period off DOX?

We re-examined more carefully the section we had stained for Ki67 and RB. However, we found no evidence of islands of cells in which RB was not re-expressed or Ki67 expression not silenced. To address this point, we added a sentence in the revised text to highlight this point.

**Minor issues**

p5 line 101 - It would be helpful to state the level of knockdown (80%) in the text.

To address this point, we revised the text to mention the level of knock-down.

p5 lines 116-118 - no expression in lung/brain: can this be shown? Where exactly? What about expression in retina?

To address the point raised by the reviewer, we generated new sh*Rb* and control mice and examined GFP expression in the lung, brain, and eye, in addition to the previous tissues we provided images for in the initial supplemental figure. These data show that, compared to the liver, intestine, and small intestine, there is minimal GFP expression in the new tissues analyzed: weak expression seems dispersed throughout the lung lobes (the IHC data discussed above suggest that some of the signal is likely to come from the bronchiolar epithelium, including club cells), while concentrated weak signal seems present in the olfactory bulb region of the brain and not in the bulk region of the brain itself. We also observed dispersed weak GFP expression in the eye. Immunostaining for GFP on eye sections showed expression in some cell types in the retina. These findings (shown in **new Supplementary Fig. S2B,C**) support our initial observation that the sh*Rb* system may not be expressed strongly in these tissues.

In addition, the authors state that low expression in the lung is due to low level expression of the ROSA locus that drives the M2rtTA in this tissue. But a lung-specific-rtTA may efficiently induce shRNA-Rb in the lung - allowing analysis of Rb loss in lung cancer and direct comparison (in future studies) with Ref. 18.

We agree with the Reviewer. As mentioned above, we are interested in lung cancer and this would be one way to bypass the low expression from the *Rosa26* promoter in the lungs. Our new immunohistochemistry data shows that also in our current mouse model efficient RB knock-down in all epithelial cells (including club cells) in the lung can be achieved, although GFP/rtTA expression levels are low. To address this point, a sentence in the Discussion now mentions examining different cancer types in the future.

Is low delivery of DOX to the brain due to the blood brain barrier (BBB)? Can this be resolved? These two issues may be briefly discussed.

To address this point, we added some text in the Discussion and a reference is now in the main text. Doxycycline can pass the BBB but probably not efficiently enough to activate the transgene (possibly 5 times less as suggested by this study: [2], with faster clearance in mice than in humans). Ways to enhance doxycycline delivery include dysbaric exposure, which increases BBB permeability to tetracycline [3] or direct injection of doxycycline into the CNS.

Fig. 5B - arrows pointing at tumors should be added.

To address this point, we added arrows to the figure in the revised version of the manuscript.

P9 235. "a normal cell cycle" - what's "normal"? Define/ rephrase.

P10 267 "including compared" - rephrase.

Fig S7 title "phenotypes of shRb mice UP RB -re-expression" - change UP to AFTER?

To address this point, we rephrased these sentences in the revised manuscript.

Fig S4B - what happens to the lens/cataract following re-expression of Rb?

This is an interesting question. We would not expect these defects to disappear upon RB re-expression because the lens does not regenerate naturally in aging mice but we had not specifically examined this phenotype in the rescue animals. To address the point raised by the Reviewer, we examined images of mouse eyes after RB re-introduction and cataract was still present, which was confirmed in eye sections (**new Supplementary Fig. S8C-D**).

Reviewer #1 (Significance (Required)):

See above.

**Reviewer #2 (Evidence, reproducibility and clarity (Required)):**

**Summary:**

Doan and colleagues present here a new mouse model which expresses a short hairpin against the RB tumor suppressor from a doxycycline-inducible promoter in the Col1a1 locus. Using this model, they demonstrate that in vivo knockdown causes defects in multiple organs. These defects included tumor growth in pituitaries and thyroids, which is also seen in Rb+/- mice or chimeric Rb-/- mice. They then provide evidence that re-establishment of RB expression by withdrawing the doxycycline treatment again blocks proliferation of the pituitary tumors.

**Major comments:**

Overall, this is a very well-written paper. The results look robust and reproducible, and in most experiments, the appropriate controls were included. The conclusions are supported by the data and the authors are not overselling their data. However ,the analysis of the mouse model could have been done in more detail to better characterize and understand the lesions induced by knocking down and re-expressing Rb.

We thank the Reviewer for this fair assessment of our work. Our goal was to describe this new model rigorously. Future experiments using sh*Rb* mice in different organs and contexts will be critical to better understand the molecular and cellular consequences of RB loss and re-introduction *in vivo*.

*Specific points:**

- In figure 2 the authors describe multiple phenotypes seen after doxycycline treatment. These include ageing-like phenotypes (kyphosis, greying fur) and enlargement of intestinal organs and inflammation. Are these phenotypes consistent with other mouse models of RB loss? What are the underlying mechanisms? Could off-target effects of the shRB construct (partly) explain those phenotypes? –

The Reviewer is raising an interesting point. Overall, the phenotypes described in the sh*Rb* model are very similar to what has been observed or described in *Rb* knockout mice, although for some of these phenotypes, previous studies did not necessarily provide a detailed description. To address the point raised by the Reviewer, we now provide a comparison between the phenotypes described in our study and phenotypes described for *Rb* mutant mice in previous studies (**new Supplementary Table S3**).

Following up on this: when I looked at the "organ enlargements" described in Figure 2D it seems that the intestines of the shRB mice are overfilled with food. Do these mice have problems with intestinal absorption and gut motility? Previous studies suggested that RB is important for differentiation of intestinal epithelium (Chong et al. Nature 2009); does this play a role here? Microscopic analysis of the intestines would be required to at least provide some basic insight. Could the intestinal lesions explain the weight loss observed in mice where Rb was knocked down?

Our first thought was that these phenotypes were simply a sign that sh*Rb* mice get sick with time, but the Reviewer brings up a good point. To address this point, we analyzed histology images from control and mutant intestines. However, we did not observe a difference in histological sections between 18w+ dox and control intestines, as described before [4], suggesting that intestinal defects may not be the cause of the weight loss.

The authors provide clear arguments why they chose to knock down RB for only 6 weeks in the rescue experiments. But even though they show increased Ki67 staining at 6 weeks, it's not clear whether at that stage the pituitaries are still preneoplastic, or already have developed a pituitary neoplasm. It would be important to know the status of the pituitary tissue at 6 weeks through histological analysis performed by an experienced pathologist.

It would be misleading to characterize the pituitary lesions at these early time points as adenocarcinomas, but previous studies call them adenomas. To address the point raised by the Reviewer, we worked with Dr. Hannes Vogel, an experienced pathologist, to provide a description of these early pituitary gland lesions. We added a better description of these lesions in the revised text.

Following up on this: did the rescue experiment show tumor regression? Pituitary masses are shown in the RB knockdown experiment (Figure 4) but not in the rescue experiment (Figure 5). It is only briefly stated that there is no difference, referring to Figure 5B, but these macroscopic photos are very hard to judge, and may not representative; hence a proper quantification of pituitary weights and histological analysis must be shown. Furthermore, what is the degree of necrosis/apoptosis/ senescence for the experiments outline in figure 5.

- Related to this above point, it is surprising that the pituitary tumors are not smaller in the 18w-res mice than in the dox-treated condition. The authors should discuss the possible reasons.

The Reviewer is raising a similar point as Reviewer #1 (point #2). We found that re-expressing RB is not sufficient to completely prevent an increase in the size of pituitary tumors, but weight increase is lower than in mice kept on doxycycline (**new Supplementary Fig. S10A**). Our current explanation is two-fold: (1) RB re-expression takes a few weeks, and tumor proliferation and growth thus does not stop right away; (2) the cells become larger when they arrest in G0/G1 (leading to tumors occupying more volume) (quantified in **new Supplementary Fig. S10B,C**); and (3) there are larger areas of necrosis (quantified in **new Supplementary Fig. S10D,E**). To further address this point, we also performed staining for the apoptotic marker cleaved caspase 3 (CC3) on sections from pituitary tumors. We observed some CC3+ signal in necrotic areas but little-to-no signal within tumors, suggest that apoptosis is not a major consequence of RB re-introduction in this context, at least at the time points examined. We provided representative images of these images in a **new Supplementary Fig. S13A**.

**Minor comments:**

-Line 229: "... variation along PCA while PC2...". It looks like the authors mean PC1 instead of PCA here.

We corrected this mistake in the revised manuscript.

-Figure 5H: the authors conclude that the variation along PC1 is mostly caused by RB re-expression, while PC2 variation is caused by tumor progression. If that is true, why did the rescue experiment only partly inhibit tumor progression? Have the authors analyzed the PCA loadings to evaluate which specific (groups of) genes drive variation in PC1 and PC2 respectively? A more in-depth analysis could help gaining better mechanistic insight into the observed phenotypes.

As discussed above, partial tumor inhibition measured by tumor area may be misleading (larger cells, more death and necrosis) and the Ki67 levels, which become barely detectable, may be a better marker of how potent RB re-expression is to suppress the growth of these pituitary tumors. To address the point raised by the Reviewer regarding the drivers of the principal component analysis (PCA), we performed additional computational analyses of the RNA-seq data and added a **new Supplementary Table S5** to the revised manuscript with a GO term analysis of genes driving PC1 and PC2. We found that the genes driving PC1 are predominantly enriched for cell cycle regulation and repair processes, while PC2 is driven by genes involved in metabolic processes, hypoxia response and many signaling pathways (e.g., NF-kappaB, Wnt). These new data provide support to our previous conclusion that PC1 variation is caused by the direct effects of RB re-expression on cell cycle progression while PC2 is more related to general tumor progression.

Reviewer #2 (Significance (Required)):

The authors present here a new mouse model in which RB can be reversibly depleted using a doxycycline-inducible shRNA. This allowed the authors to study investigate if re-expression of RB loss-induced tumors. Interestingly the proliferation of pituitary tumor cells strongly depends on the RB status. In a previous study, others already showed the effect of re-expressing RB in KRAS/P53/Rb mutant mouse lung adenocarcinoma [1]. The results of the present study seemingly contradict that previous work, because RB expression did not halt cancer cell proliferation in lung adenocarcinomas but in pituitary is does. However, the models are different and potential tissue specific function for RB and the different genetic context could explain the observations. Probably the tumor stage at the moment of re-expression is of critical importance as well, but unfortunately the authors have not address this in the presented model.

This is a really good point raised by the Reviewer. To address this point, we revised the Discussion to emphasize the context-dependency of the requirements for both RB loss in cancer initiation and cancer maintenance. The idea of re-expressing RB at different times during tumor development is also what we had originally in mind and what we actually did and presented in this manuscript. Unfortunately, when we re-expressed RB in late-stage tumors, we observed cell cycle arrest but were not able to study these mice for a long time because of their death from these tumors. This is why we also performed experiments with earlier-stage tumors in the last part of the manuscript. A limitation of the sh*Rb* model is that it takes 2-3 weeks to re-express RB, which limits how acutely these re-introduction experiments can be performed. It will be interesting in the future to use the sh*Rb* mice in lung cancer models and compare with the work from the Feldser lab using their XTR allele [5].

This study is of interest to the field of cancer genetics, and especially to those interested in understanding the molecular actions of tumor suppressor proteins, including RB/E2F. For researchers working with genetic mouse models, the approaches in this study could be of interest, although the approach is not entirely new. For example, APC knockdown and re-expression has been studied in a similar model [2].

It is true that the use of inducible shRNA in transgenic mice is not novel (Dr. Dow is a co-author on our study and was the lead author in the APC study [6]. We believe that it is still important to develop and characterize different models for different tumor suppressors. These different models may provide novel insights into the different mechanisms of action of tumor suppressors in different cancer types. We revised the Discussion to address this point.

It is a bit difficult to see how these results could be translated to human cancer patients. And although of interest and well-performed, this study does not provide a completely new concept or idea in my view, since it expected that re-expression of Rb will stop cell proliferation.

In terms of human cancer relevance, we note that human pituitary tumors show silencing of *RB1* by DNA methylation (30-35% of cases) as well as *CDKN2A* coding for p16^INK4A^ (60-70% of cases) [7-9], indicating that pituitary tumors in RB-deficient mice are a relevant model for this human cancer type. We also want to emphasize that a main goal for developing sh*Rb* mice is to discover key mechanisms of action of RB. Studies in mouse pituitary and thyroid tumors are relevant to other cancer types, as shown before. For example, the demonstration that E2F is a key mediator of cancer phenotypes downstream of loss of RB was first made in mouse pituitary tumors [10]. That said, we fully agree with the Reviewer that our studies will be even more relevant in the context of other cancer types, including lung and breast cancer. We hope that the Reviewer will agree with us that these studies belong to future manuscripts.

It was also expected in the Feldser lab study [5] that once cells have arrested upon RB re-introduction, they would remain arrested. However, cancer cells with RB re-introduction only stopped cycling transiently, so one could argue that our “expected” observation may not have been 100% expected and that it is important to test this model in several cancer contexts.

To address these points, we made sure in the revised text to discuss this more thoroughly.

My field of expertise is cell cycle control, E2F/RB, cell biology, transcriptomics, and genetic mouse cancer models.

[1] Walter DM, Yates TJ, Ruiz-Torres M, Kim-Kiselak C, Gudiel AA, Deshpande C, et al. RB constrains lineage fidelity and multiple stages of tumour progression and metastasis. Nature. 2019;569(7756):423-7. doi: 10.1038/s41586-019-1172-9.s

[2] Dow LE, O'Rourke KP, Simon J, Tschaharganeh DF, van Es JH, Clevers H, et al. Apc Restoration Promotes Cellular Differentiation and Reestablishes Crypt Homeostasis in Colorectal Cancer. Cell. 2015;161(7):1539-52. Epub 2015/06/20. doi:649 10.1016/j.cell.2015.05.033.

**Referee Cross-commenting**

I agree also with the other reviewer comments. Similar to reviewer 3 I believe that performing additional developmental studies are beyond the scope of this study. Furthermore, studying RB function during development is complex because you have to take into account both placental and embryonic specific function of RB. These studies would take at least another 2 years to perform.

We had begun to investigate how to knock-down RB during embryonic development, as discussed in response to the comment from Reviewer #1 but decided not to pursue these experiments because the complex phenotypes observed in pilot experiments indicate that such developmental studies will require various experimental approaches and assays, and we appreciate that the Reviewer also thinks that these experiments are beyond the scope of this study.

**Reviewer #3 (Evidence, reproducibility and clarity (Required)):**

This manuscript uses a newly developed inducible shRNA mouse model to study the effects of reversible RB depletion and reactivation in a broad array of tissues. The authors find that RB depletion induces several phenotypes associated with aging and inflammation. Similar to Rb knockout mice, mice expressing the inducible shRb transgene also develop pituitary and thyroid tumors. This result confirms the accuracy of this model for studying the impact of RB loss. An advantage of this model, however, is that RB can be reactivated to study how this impacts phenotypes, including tumor progression. The authors find that re-expression of RB results in a stabilization of phenotypes, induces a sustained inhibition of cell proliferation in pituitary adenomas, and prevents thyroid tumorigenesis. The findings are convincing and well controlled.

Based on these findings the authors conclude that continuous RB loss is required for the maintenance of tumor phenotypes initiated by loss of RB. This conclusion is in contrast to a previously reported study using a genetic recombination mouse model in which Rb re-expression resulted in only a transient inhibition of cancer cell proliferation in a K-ras mediated lung tumors due to upregulation of CDK2 activity. Thus, the above conclusion is valid if considering tumors "initiated by loss of RB" but this is not necessarily the case for all tumor types involving loss of RB function. This context-dependent effect of RB reactivation should be further discussed, particularly in light of understanding which cancers might respond to CDK inhibition and which might quickly develop resistance. Otherwise, the methods are presented clearly and experiments are adequately replicated.

We fully agree with these comments from the Reviewer. As discussed above in response to the comment from Reviewer #2, to address this point, we made sure in the revised discussion to emphasize the context-dependency of the requirements for both RB loss in cancer initiation and cancer maintenance.

**Some minor comments.**

1) In the manuscript text, line 104, the authors state that the shRb5-4 mESC clone was used to generate transgenic mice because, in part, it had a normal karyotype. However, Figure S1D indicates that clone 4 is aneuploid because it is marked with a star (clone 4*). Doesn't shRb5-4 indicate the shRb5 transgene, clone 4? Please explain/address this discrepancy.

We apologized for this mistake, we meant to write “euploid” for clones 2 and 4. We corrected this mistake in the revised version of the manuscript.

2) The phenotype description of the shRb expressing mice on line 134 is unclear. Do you mean 4 out of 4 mice expressing shRb had a scruffy greying hair coat and....

We thank the Reviewer for picking this other mistake, we rewrote this sentence, 4/4 sh*Rb* mice showed these phenotypes.

3) Can the authors speculate as to why loss of RB results in phenotypes associated with aging? Have they looked at senescence in affected tissues?

We were struck when we analyzed the phenotypes of these mice carefully how they resemble some aging phenotypes. It is very likely that some (or most) of these phenotypes are due to hormonal imbalances (with tumors growing in the pituitary and the thyroid glands). In support of this idea, similar phenotypes have been described (although not in great detail) in *Rb^+/-^* mice and mice in which *Rb* was deleted specifically in the pituitary gland [11]. We did not examine senescence in tissues and organs in these mice and hope that the Reviewer will agree with us that studies of cell autonomous and non-cell autonomous mechanisms of organismal and cellular aging are beyond the scope of this first description of sh*Rb* mice.

This point is also similar to the first point raised by Reviewer #2, and we now provide a comparison between the phenotypes described in our study and phenotypes described for *Rb* mutant mice in previous studies to highlight some of these phenotypes (**new Supplementary Table S3**).

Reviewer #3 (Significance (Required)):

The significance of this work includes the development of a new mouse model of RB deficiency that complements existing Rb mouse models. An advantage of this new mouse model is the ability reactivate RB to study how therapeutic interventions that attempt to reactivate RB, such as CDK inhibitors, impact cancer progression long term. The authors show that reactivation of RB in at least some contexts can have long-term benefits in preventing cancer progression. This adds to the existing literature that reactivation of RB using various strategies can be an effective therapeutic approach in some, but not all settings. This model will be useful in the future in helping to define the contexts in which RB reactivation is beneficial. As a researcher in the E2F-RB field that uses mouse models, I found this study elegant and well done despite having to overcome some technical issues, such as the slow reactivation of RB.

**Referee Cross-commenting**

I agree with the other reviewer's comments although I think asking the authors to perform the embryonic development studies may be asking too much. We all seem to agree it is a solid study.

We thank the Reviewer for this positive view of our work. As discussed above, we are interested in the possible use of sh*Rb* mice in mouse embryos, but this may be the focus of a different manuscript because the complex phenotypes observed in pilot experiments indicate that such developmental studies will require various experimental approaches and assays and we appreciate that the Reviewer has the same opinion.

**Reviewer #4 (Evidence, reproducibility and clarity (Required)):**

The retinoblastoma tumor suppressor protein, RB, plays a major role in constraining both cancer development and cancer progression in multiple tissue types. Despite clinical observations supporting the concept that RB loss promotes tumor phenotypes, significant gaps remain in discerning the overarching impact of RB status as a function of tumor progression. The present study describes a new mouse model, generated toward the goal of understanding the impact of RB loss and subsequent reactivation in vivo. Key findings of the study demonstrate that the new regulatable model systems develop the expected tumor profile after RB loss (e.g., thyroid and pituitary tumors), and that subsequent RB re-expression induces cell cycle arrest in pituitary adenomas. The collective observations provide compelling evidence that RB depletion is required for the continuous growth of tumors initiated by RB loss, thus providing new impetus for developing strategies beyond CDK4/ inhibitors to re-engage RB pathway function.

**Critique:**

This is a well conducted study that reports generation of an elegant new model system for studying RB function in vivo. The data shown generally support the stated conclusions, and the study will be of significant impact for the cell cycle and tumor suppressor field. Addressing the following minor concerns would further increase the impact of the findings.

We thank the Reviewer for this positive view of our work.

1. It would be of benefit to determine the impact of RB depletion and reintroduction on potential compensatory pathways (e.g., p107 and p130 expression), and to report the impact on individual activator E2F family members.

This is a good point raised by the Reviewer, as functional overlap and compensation are well known to exist between RB family members. Over the years, we have not been successful in establishing reliable immunostaining protocols for p107 and p130 unfortunately. To address the point raised by the Reviewer, we performed RT-qPCR for *p107* (*Rbl1*) and *p130* (*Rbl2*) and genes coding for activator E2Fs in pituitary tumor samples following RB re-introduction. As expected, we found decreased levels of these genes in tumors 2 weeks after doxycycline removal (**new Supplementary Fig. S6**).

2. The interferon response pathway appears to be significantly altered as a function of RB status (Figure 5). It would be of interest to determine or speculate the impact of this finding as related to observed tumor responses.

There is emerging evidence that the RB pathway is connected with immune pathways, including the interferon pathway (see Knudsen et al., 2021, as a recent example [12]). We made sure to include the proper references in the revised manuscript. We were initially intrigued by this observation in our RNA-seq and wondered whether re-introduction of RB may affect the microenvironment of pituitary tumors, especially immune cells. Our first analysis of the RNA-seq data using CIBERSORT to deconvolute possible signals from immune cells did not reveal any striking difference and was largely inconclusive, and we decided not to include this analysis in the manuscript. To address the point raised by the Reviewer, we performed immunostaining for CD45^+^ hematopoietic cells on sections from pituitary tumors after RB re-introduction. These data show a trend towards an increasing infiltration by immune cells in the tumor area upon Rb reintroduction (**new Supplementary Fig. S12**), which is likely the reason for the altered interferon response pathway found in our RNA-seq data. We do not know the contribution of these immune cells to tumor inhibition, but this type studies will be the focus of future work

3. In addition to above, the observation of EMT related gene expression changes could be of significant relevance. It will be important to determine whether cell fate changes or markers of plasticity occur as a result of changing RB status.

We and others have connected RB to cell plasticity and cell fate decisions (e.g., [5] or [13]). The comment by the Reviewer encompasses a broad range of cellular and molecular processes and it is difficult for us to think about the best way to characterize these processes in the current study. Thus, to address the point raised by the Reviewer, we revised the Discussion section and mentioned the possible use of this sh*Rb* model in the future to address how RB controls cell fate decisions in various contexts.

Reviewer #4 (Significance (Required)):

see above.

**Selected References:**

1.	Fischer M. Census and evaluation of p53 target genes. Oncogene. 2017;36(28):3943-56. Epub 2017/03/14. doi: 10.1038/onc.2016.502. PubMed PMID: 28288132; PubMed Central PMCID: PMCPMC5511239.

2.	Lucchetti J, Fracasso C, Balducci C, Passoni A, Forloni G, Salmona M, et al. Plasma and Brain Concentrations of Doxycycline after Single and Repeated Doses in Wild-Type and APP23 Mice. J Pharmacol Exp Ther. 2019;368(1):32-40. Epub 2018/11/07. doi: 10.1124/jpet.118.252064. PubMed PMID: 30396916.

3.	Chryassanthou C, Graber B, Mendelson S, Goldstein G. Increased blood-brain barrier permeability to tetracycline in rabbits under dysbaric conditions. Undersea Biomed Res. 1979;6(4):319-28. Epub 1979/12/01. PubMed PMID: 538860.

4.	Haigis K, Sage J, Glickman J, Shafer S, Jacks T. The related retinoblastoma (pRb) and p130 proteins cooperate to regulate homeostasis in the intestinal epithelium. J Biol Chem. 2006;281(1):638-47. Epub 2005/11/01. doi: 10.1074/jbc.M509053200. PubMed PMID: 16258171.

5.	Walter DM, Yates TJ, Ruiz-Torres M, Kim-Kiselak C, Gudiel AA, Deshpande C, et al. RB constrains lineage fidelity and multiple stages of tumour progression and metastasis. Nature. 2019;569(7756):423-7. doi: 10.1038/s41586-019-1172-9. PubMed PMID: 31043741; PubMed Central PMCID: PMCPMC6522292.

6.	Dow LE, O'Rourke KP, Simon J, Tschaharganeh DF, van Es JH, Clevers H, et al. Apc Restoration Promotes Cellular Differentiation and Reestablishes Crypt Homeostasis in Colorectal Cancer. Cell. 2015;161(7):1539-52. Epub 2015/06/20. doi: 10.1016/j.cell.2015.05.033. PubMed PMID: 26091037; PubMed Central PMCID: PMCPMC4475279.

7.	Ogino A, Yoshino A, Katayama Y, Watanabe T, Ota T, Komine C, et al. The p15(INK4b)/p16(INK4a)/RB1 pathway is frequently deregulated in human pituitary adenomas. J Neuropathol Exp Neurol. 2005;64(5):398-403. Epub 2005/05/17. doi: 10.1093/jnen/64.5.398. PubMed PMID: 15892297.

8.	Simpson DJ, Hibberts NA, McNicol AM, Clayton RN, Farrell WE. Loss of pRb expression in pituitary adenomas is associated with methylation of the RB1 CpG island. Cancer Res. 2000;60(5):1211-6. Epub 2000/03/23. PubMed PMID: 10728677.

9.	Yoshino A, Katayama Y, Ogino A, Watanabe T, Yachi K, Ohta T, et al. Promoter hypermethylation profile of cell cycle regulator genes in pituitary adenomas. J Neurooncol. 2007;83(2):153-62. Epub 2007/01/12. doi: 10.1007/s11060-006-9316-9. PubMed PMID: 17216555.

10.	Yamasaki L, Bronson R, Williams BO, Dyson NJ, Harlow E, Jacks T. Loss of E2F-1 reduces tumorigenesis and extends the lifespan of Rb1(+/-)mice. Nat Genet. 1998;18(4):360-4. Epub 1998/04/16. doi: 10.1038/ng0498-360. PubMed PMID: 9537419.

11.	Vooijs M, van der Valk M, te Riele H, Berns A. Flp-mediated tissue-specific inactivation of the retinoblastoma tumor suppressor gene in the mouse. Oncogene. 1998;17(1):1-12. Epub 1998/07/22. doi: 10.1038/sj.onc.1202169. PubMed PMID: 9671308.

12.	Knudsen ES, Kumarasamy V, Chung S, Ruiz A, Vail P, Tzetzo S, et al. Targeting dual signalling pathways in concert with immune checkpoints for the treatment of pancreatic cancer. Gut. 2021;70(1):127-38. Epub 2020/05/20. doi: 10.1136/gutjnl-2020-321000. PubMed PMID: 32424005; PubMed Central PMCID: PMCPMC7671951.

13.	Kareta MS, Gorges LL, Hafeez S, Benayoun BA, Marro S, Zmoos AF, et al. Inhibition of pluripotency networks by the Rb tumor suppressor restricts reprogramming and tumorigenesis. Cell Stem Cell. 2015;16(1):39-50. Epub 2014/12/04. doi: 10.1016/j.stem.2014.10.019. PubMed PMID: 25467916; PubMed Central PMCID: PMCPMC4389904.
